# Efficacy and Safety of Lebrikizumab in Adults with Moderate-to-Severe Atopic Dermatitis: A Systematic Review and Meta-Analysis

**DOI:** 10.3390/jcm15051737

**Published:** 2026-02-25

**Authors:** Oscar M. Lopez-Mallama, Raul Sandoval-Ato, Pedro Ruiz Vega, Hady Keita, Gerson Diaz-Gonzales, Oriana Rivera-Lozada, Joshuan J. Barboza

**Affiliations:** 1 Institución Universitaria Antonio Jose Camacho, Universidad del Valle, Cali 760046, Colombia; omarinolopez@admon.uniajc.edu.co; 2 Escuela de Medicina, Universidad Privada Antenor Orrego, Trujillo 13008, Peru; genrhraul@gmail.com (R.S.-A.); pedroruizvega97@gmail.com (P.R.V.); 3 Escuela de Medicina, Universidad de la Sierra Sur, Oaxaca 70805, Mexico; hadykeith@yahoo.fr; 4 Escuela de Medicina, Universidad San Ignacio de Loyola, Lima 15024, Peru; gdiazc@usil.edu.pe; 5 Vicerrectorado de Investigación, Universidad Señor de Sipán, Chiclayo 14001, Peru; riveraoriana@uss.edu.pe; 6 Escuela de Medicina, Universidad Señor de Sipán, Chiclayo 14001, Peru

**Keywords:** systematic review, meta-analysis, lebrikizumab, atopic dermatitis

## Abstract

**Background**: Atopic dermatitis (AD) is a chronic inflammatory skin disease associated with substantial symptom burden and impaired quality of life. Lebrikizumab, a monoclonal antibody targeting interleukin-13, has emerged as a therapeutic option for patients with moderate-to-severe AD; however, a comprehensive synthesis of its efficacy and safety is required. **Methods**: We conducted a systematic review and meta-analysis of randomized controlled trials comparing lebrikizumab with placebo in patients with moderate-to-severe AD. Searches were performed across major databases and trial registries. The primary outcome was achievement of a 50% improvement in the Eczema Area and Severity Index (EASI-50). Secondary efficacy outcomes included EASI-75, EASI-90, and improvement in pruritus measured by the Numeric Rating Scale (NRS). Safety was assessed through a quantitative meta-analysis of treatment-emergent adverse events (TEAEs) when extractable arm-level data were available. Random-effects models were applied, and certainty of evidence was evaluated using the GRADE framework. **Results**: Twelve randomized controlled trials were included. Lebrikizumab significantly increased the likelihood of achieving EASI-50 (RR 1.51, 95% CI 1.20–1.89), EASI-75 (RR 1.78, 95% CI 1.43–2.22), and EASI-90 (RR 2.26, 95% CI 1.67–3.06) compared with placebo, and was associated with clinically meaningful improvement in pruritus (RR 1.73, 95% CI 1.38–2.17). Substantial heterogeneity was observed for EASI-75 (I^2^ = 77.5%); predefined subgroup analyses based on dosing regimen and dosing frequency partially explored this variability, but residual heterogeneity persisted, leading to downgrading of the certainty of evidence for EASI-75 to low. The certainty of evidence was moderate for EASI-50 and low for EASI-90 and pruritus improvement. Six trials contributed to the quantitative safety analysis. The pooled meta-analysis showed no significant difference in the risk of treatment-emergent adverse events between lebrikizumab and placebo (RR 1.03, 95% CI 0.82–1.28), with moderate heterogeneity (I^2^ = 60.5%). Serious adverse events and treatment discontinuations were infrequently reported and could not be pooled quantitatively due to inconsistent reporting. **Conclusions**: Lebrikizumab demonstrates clinically meaningful efficacy and a favorable safety profile in patients with moderate-to-severe atopic dermatitis. However, as the available randomized evidence is predominantly derived from adult populations, the applicability of these findings to adolescents remains limited and warrants confirmation in adequately powered, adolescent-focused studies.

## 1. Introduction

Atopic dermatitis (AD) is a chronic inflammatory skin disease characterized by recurrent eczematous lesions and pruritus, with a substantial impact on quality of life; persistent physical pain and psychological exhaustion become the central problem [1]. Atopic dermatitis is a chronic inflammatory condition affecting approximately 2% to 7% of the adult population, characterized by recurrent flares of pruritus that may impair sleep quality and contribute to anxiety and depression, particularly in individuals with more severe disease [1,2,3]. Approximately 30% of patients present with moderate-to-severe atopic dermatitis, a disease severity that cannot be adequately controlled with topical therapy alone and therefore requires systemic treatment [2].

Biologically, the pathophysiology of atopic dermatitis (AD) results from a breakdown of the epidermal barrier and a deficient immune system, primarily due to type 2 inflammation [4]. Of all known cytokines, interleukin-13 (IL-13) stands out as the main culprit. This is because this cytokine not only triggers inflammation but also weakens the structural integrity of the skin by regulating the reduction in filaggrin [5,6]. This specific function of IL-13 makes it an ideal target for a more nuanced approach and the application of precision medicine.

Lebrikizumab is a high-affinity monoclonal antibody that selectively neutralizes soluble interleukin-13 (IL-13), thereby preventing activation of the IL-13Rα1/IL-4Rα signaling receptor complex and downstream type 2 inflammatory pathways [6]. IL-13 signaling is physiologically regulated by two distinct receptor subunits: IL-13Rα1, which mediates intracellular signaling, and IL-13Rα2, a high-affinity decoy receptor that lacks signaling capacity and contributes to sequestration and internalization of IL-13 [6,7]. By leaving this receptor alone, the drug allows the body to maintain its natural process of clearing excess IL-13, resulting in a pharmacological profile that stands apart from its predecessors. Importantly, lebrikizumab binds IL-13 at an epitope that does not interfere with IL-13 interaction with IL-13Rα2, thereby preserving IL-13 clearance through this decoy receptor pathway [6,7]. In contrast, other IL-13–targeting antibodies, such as tralokinumab, bind IL-13 at epitopes that overlap receptor-binding sites and prevent interaction with both IL-13Rα1 and IL-13Rα2, while dupilumab inhibits IL-13 signaling indirectly by blocking IL-4Rα, a shared receptor subunit for IL-4 and IL-13 signaling [6,7]. This mechanistic distinction may underlie differences in pharmacologic and clinical profiles among available biologic therapies for atopic dermatitis.

While the efficacy of lebrikizumab has been signaled in earlier studies, the therapeutic landscape is changing rapidly. The recent conclusion of several landmark phase 3 trials means that an isolated look at old data is no longer enough. We conducted this systematic review and meta-analysis to integrate these latest findings, offering a rigorous, updated evaluation of how lebrikizumab truly performs—both in terms of safety and clinical success—for adolescents and adults struggling with moderate-to-severe AD.

## 2. Materials and Methods

### 2.1. Study Design

This study was a systematic review with meta-analysis. The reference elements for systematic reviews and meta-analyses (PRISMA-2020) informed this review (Supplemental Table S2) [8]. We assessed studies that evaluated the efficacy of treatment with lebrikizumab in patients with atopic dermatitis. The protocol was registered in the PROSPERO database (CRD42025635083).

### 2.2. Searches

We searched PubMed/MEDLINE, Embase, Web of Science, and Scopus. Searches were performed from their inception until 14 January 2025, including key phrases, MeSH terms (PubMed), and Emtree thesauri (Scopus, Embase). Finally, a search strategy was applied to each database (Supplemental Table S1). (“Severe Atopic Dermatitis”) AND (Lebrikizumab) were the primary search phrases. There were no limitations on language or date of publication. In addition, all reference lists of relevant studies and included review articles were hand-searched for other potentially eligible trials.

### 2.3. Elegibility Criteria

All studies that met the following criteria were included in this study: Phase 2 or Phase 3 randomized controlled trials that treated adolescents and adults diagnosed with moderate-to-severe atopic dermatitis and used lebrikizumab (125 μg to 400 μg maximum dose, twice daily) as the intervention compared to placebo. Conference abstracts, systematic reviews, narrative reviews, case reports and series, and letters to the editor were excluded.

### 2.4. Outcomes

The primary outcome was the proportion of patients achieving a 50% improvement in the Eczema Area and Severity Index (EASI-50). Secondary efficacy outcomes included achievement of 75% improvement in EASI (EASI-75) and 90% improvement in EASI (EASI-90), as well as improvement in pruritus assessed using the Pruritus Numeric Rating Scale (NRS), an 11-point patient-reported scale.

The Eczema Area and Severity Index (EASI) is a composite measure ranging from 0 to 72, with higher scores indicating more severe and/or extensive atopic dermatitis. Treatment responses were defined as EASI-50 (≥50% reduction from baseline), EASI-75 (≥75% reduction from baseline), and EASI-90 (≥90% reduction from baseline) in accordance with standard definitions used in randomized controlled trials [9].

Pruritus severity was assessed using the Pruritus Numeric Rating Scale (NRS), an 11-point scale in which patients rate the intensity of their worst itch during the previous 24 h, with scores ranging from 0 (“no itch”) to 10 (“worst itch imaginable”) [10].

As a safety outcome, the occurrence of treatment-emergent adverse events (TEAEs) was evaluated. TEAEs were defined as any adverse events reported after initiation of the study intervention, irrespective of severity or presumed causality, as reported in the included randomized controlled trials. Only studies providing extractable arm-level data for TEAEs in both lebrikizumab and control groups were included in the quantitative synthesis.

### 2.5. Data Extraction

After the electronic searches, the results were compiled in a single library, and duplicates were eliminated. Then, independently and blinded, the first screening step was performed, evaluating the titles and abstracts and applying the inclusion and exclusion criteria to each result reviewed through the Rayyan platform. The studies included after this phase were searched and analyzed in full text, and then a new screening process was carried out, justifying the inclusion and exclusion criteria. After this process, the eligible studies were included in the systematic review, and data extraction began. A third review author (JJB) was consulted in cases of disagreement.

Data were extracted from each study individually and blinded using a pre-prepared Excel spreadsheet format. For each analysis, data were extracted on the author, year of publication, country, type of study, number of participants per intervention arm, selection criteria, description of intervention and control, and primary and secondary outcomes.

### 2.6. Risk of Bias Assessment

The risk of bias (RoB) was independently assessed using the RoB 2.0 tool. Disagreements were resolved through discussion with a third author (JJB). RoB for each domain and study was described as low, with some concerns, or high for randomized controlled trials (RCTs).

### 2.7. Data Synthesis

Random-effects models using the inverse variance method were applied for all meta-analyses comparing lebrikizumab with placebo across primary and secondary outcomes in patients with atopic dermatitis. Between-study variance (τ^2^) was estimated using the Paule–Mandel method. Treatment effects for dichotomous outcomes were expressed as relative risks (RRs) with corresponding 95% confidence intervals (CIs). Continuity corrections were applied when zero events occurred in one or both study arms. When more than five studies were included in a pooled analysis, the Hartung–Knapp adjustment was used to provide more conservative confidence intervals [11].

Statistical heterogeneity was assessed using the I^2^ statistic, with values interpreted as low (<30%), moderate (30–60%), or substantial (>60%) heterogeneity. In the presence of substantial heterogeneity, predefined subgroup analyses were conducted for the primary outcome (EASI-75) to explore potential sources of clinical variability. Subgroups were defined according to dosing regimen (125 mg versus 250 mg; Q2W versus Q4W) and follow-up duration (induction phase at 12–16 weeks versus maintenance phase at ≥52 weeks). Subgroup analyses were performed only when sufficient data were available, and pooled estimates were calculated separately within each subgroup.

Sensitivity analyses were conducted using fixed-effect models and the Mantel–Haenszel method to assess the robustness of the pooled estimates. Assessment of publication bias using funnel plots and Egger’s regression test was planned a priori; however, these analyses were not performed because fewer than ten studies were available for the relevant outcomes, in accordance with methodological recommendations. All analyses were performed using the metabin function of the meta package in R (version 3.5.1; www.r-project.org).

### 2.8. GRADE Quality of Evidence

The GRADE methodology assessed the evidence’s certainty and the intervention’s degree of recommendation for all outcomes. GRADE is based on its domains, including the risk of bias, inconsistency, indirectness, imprecision, and publication bias, which were evaluated systematically. The certainty of the evidence was determined for each outcome and described in the summary of results (SoF) tables, which were created using the online software GRADEpro GDT (https://www.gradepro.org) [12].

## 3. Results

### 3.1. Selection of Studies

**Figure 1:**
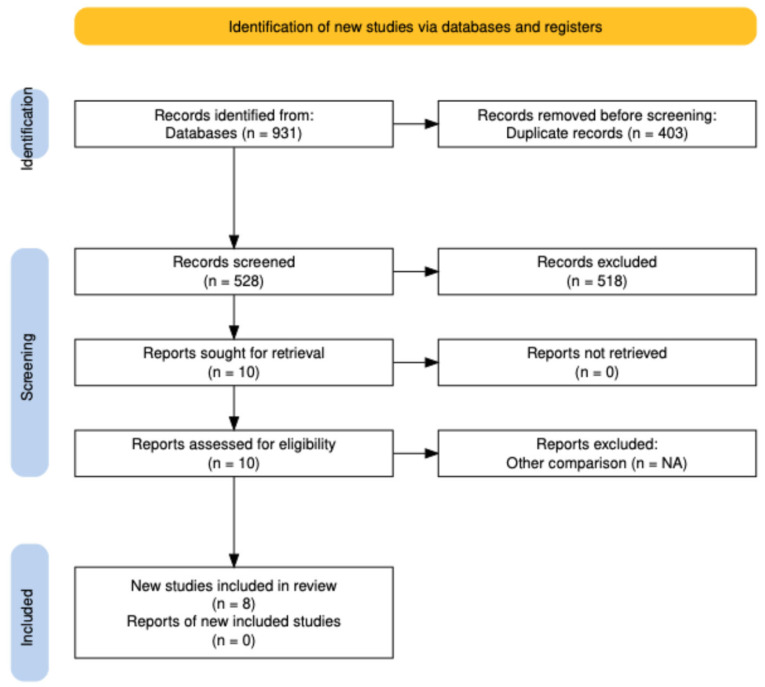
PRISMA Selection of Studies.

A total of 931 articles were identified in four databases; 403 duplicate articles were removed. Of 528 screened abstracts, 428 were excluded. Thus, ten full-text studies were assessed for eligibility, and two were excluded. Finally, eight studies (RCTs; n = 8) were included for qualitative and quantitative analyses [4,9,13,14,15,16,17,18] (Figure 1).

### 3.2. Characteristics of Included Studies

The clinical trials included in this review spanned the years 2018 to 2024. Most of the studies were conducted in phase 3, while some, such as those by Emma Guttman-Yassky et al. (2020) [13] and Eric L. Simpson et al. (2018) [16], were conducted in phases 2b and 2, respectively. The studies involved 37 and 171 research centers across North America, Europe, Asia-Pacific, and the United States. Some, such as those by Andrew Blauvelt et al. (2023) [4] and Eric Simpson et al. (2025) [18], covered multinational networks of up to 171 and 100 sites, respectively, while others, such as those by Emma Guttman-Yassky et al. (2020) [13] and Akio Tanaka et al. (2024) [15], were conducted at a more limited number of centers (Table 1).

The studied population consisted of patients with chronic atopic dermatitis, defined by an EASI (Eczema Area and Severity Index) score ≥ 16, an Investigator’s Global Assessment (IGA) score ≥ 3, and involvement of at least 10% of the body surface area (BSA). Additionally, participants frequently presented comorbidities such as asthma and allergic rhinitis, while some also reported facial dermatitis and conjunctivitis.

Inclusion criteria focused on patients with moderate-to-severe atopic dermatitis who had not responded adequately to prior topical treatments. In contrast, those with concomitant skin conditions such as T-cell lymphoma or allergic contact dermatitis were excluded (Table 2).

Follow-up periods ranged from 12 to 52 weeks, with some studies adding a safety follow-up of up to 10 weeks. Dropout rates during follow-up were low in most trials. Treatment adherence was consistently high, exceeding 77% in all studies. Clinical evaluations highlighted significant improvements across various indicators. In the Investigator’s Global Assessment (IGA), many patients achieved reductions of at least two points, reaching final scores of 0 or 1. In the Eczema Area and Severity Index (EASI), reductions exceeding 75% (EASI-75) and even 90% (EASI-90) were observed, indicating substantial improvement in the severity of dermatitis (Table 3).

### 3.3. Risk of Bias

**Figure 2:**
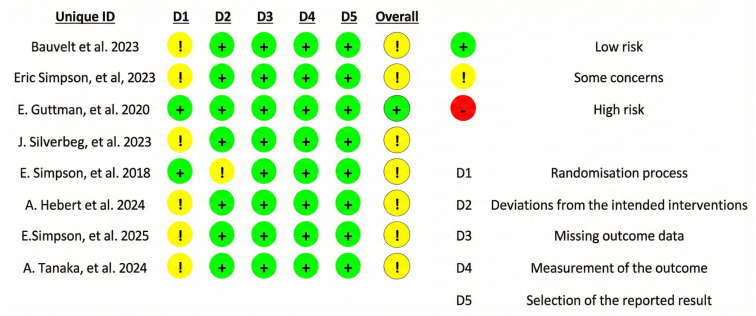
Risk of bias assessment [4,9,13,14,15,16,17,18].

The risk of bias was assessed for all studies included in this systematic review using the RoB 2.0 tool, evaluating the five predefined domains. Across all studies, Domain 1 (bias arising from the randomization process) was consistently rated as having “some concerns.” This was primarily due to incomplete details on allocation concealment methods or baseline imbalances that were not adequately addressed in the reports. Overall, the studies demonstrated a generally reliable methodological framework, with most concerns stemming from reporting deficiencies in the randomization process (Figure 2).

### 3.4. Effects of Lebrikizumab on Primary and Secondary Outcomes

**Figure 3:**
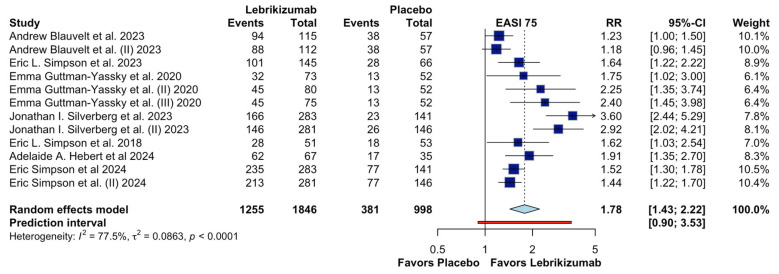
Effects of Lebrikizumab on EASI 75 [4,9,13,14,16,17,18].

Figure 3 shows the results of the meta-analysis evaluating the effect of lebrikizumab compared with placebo on achieving a 75% improvement in the Eczema Area and Severity Index (EASI-75) in patients with moderate-to-severe atopic dermatitis. A total of 12 randomized controlled trials were included. Overall, lebrikizumab was associated with a significantly higher likelihood of achieving EASI-75 compared with placebo (RR 1.78, 95% CI 1.43–2.22). However, substantial heterogeneity was observed across studies (I^2^ = 77.5%, τ^2^ = 0.0863, *p* < 0.0001), indicating considerable variability in effect estimates.

**Figure 4:**
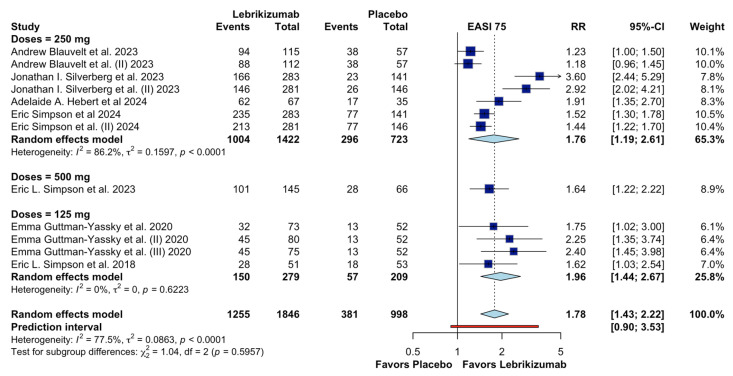
Subgroup analysis by doses on EASI 75 [4,9,13,14,16,17,18].

Given this level of heterogeneity, predefined subgroup analyses were conducted to explore potential sources of clinical variability (Figure 4). Subgroup analyses according to dosing regimens demonstrated that lebrikizumab administered at 250 mg was associated with a significant improvement in EASI-75 (RR 1.76, 95% CI 1.19–2.61), albeit with substantial residual heterogeneity (I^2^ = 86.2%), whereas the 125 mg regimen showed a comparable effect size with no observed heterogeneity (RR 1.96, 95% CI 1.44–2.67; I^2^ = 0%). A single study evaluating a 500 mg dose also favored lebrikizumab (RR 1.64, 95% CI 1.22–2.22). Despite these differences in heterogeneity patterns, the test for subgroup differences by dose was not statistically significant (χ^2^ = 1.04, df = 2, *p* = 0.5957), suggesting no clear effect modification by dose.

**Figure 5:**
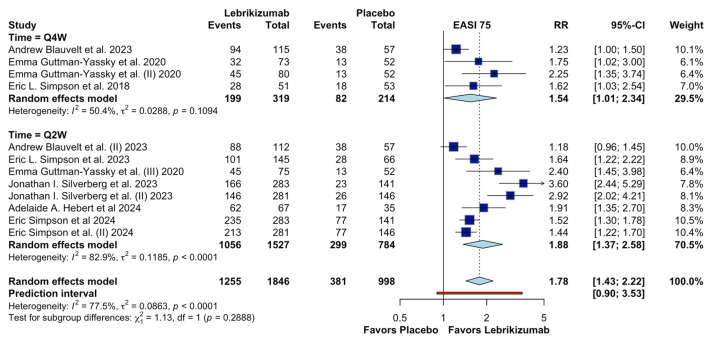
Subgroup analysis by time on EASI 75 [4,9,13,14,16,17,18].

Additional subgroup analyses based on dosing frequency (time-point) showed that both regimens favored lebrikizumab (Figure 5). Trials using a Q4W regimen demonstrated a pooled RR of 1.54 (95% CI 1.01–2.34) with moderate heterogeneity (I^2^ = 50.4%), whereas Q2W regimens were associated with a pooled RR of 1.88 (95% CI 1.37–2.58) and substantial heterogeneity (I^2^ = 82.9%). The test for subgroup differences between Q2W and Q4W regimens was not statistically significant (χ^2^ = 1.13, df = 1, *p* = 0.2888), indicating that dosing frequency did not fully explain the observed heterogeneity.

The prediction interval for the overall EASI-75 analysis was wide (RR 0.90–3.53), further reflecting between-study variability and uncertainty in the magnitude of effect across different clinical settings. Taken together, although subgroup analyses by dose and dosing frequency partially elucidated patterns of variability, substantial heterogeneity persisted, supporting cautious interpretation of the pooled effect estimate. Consequently, the certainty of evidence for the EASI-75 outcome was downgraded for inconsistency in the GRADE assessment.

**Figure 6:**
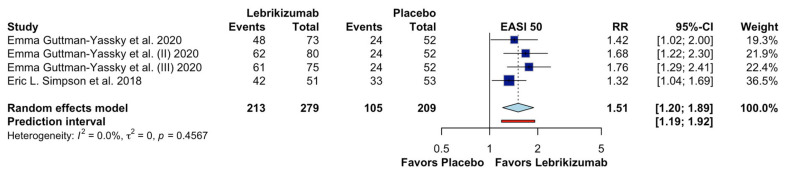
Effects of Lebrikizumab on EASI 50 [13,16].

Figure 6 presents the results of the meta-analysis for the outcome 50% improvement in the Eczema Area and Severity Index (EASI-50) among patients with moderate to severe atopic dermatitis. Four randomized controlled trials (RCTs) were included, with 279 patients in the lebrikizumab group and 209 in the placebo group. We note that lebrikizumab likely produces a slight increase in EASI-50 (RR 1.51; 95% CI 1.20-1.89; CoE Moderate), indicating that lebrikizumab-treated patients were approximately 51% more likely to achieve EASI-50 compared to those receiving placebo. In contrast to the EASI-75 result, no substantial heterogeneity was observed across studies, with an I^2^ value of 0% and a τ^2^ of 0, suggesting consistency in effect estimates across trials.

**Figure 7:**
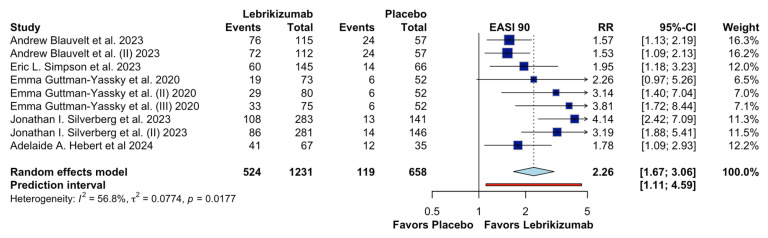
Effects of Lebrikizumab on EASI 75 [4,9,13,14,17].

Figure 7 evaluates the meta-analysis for the outcome 90% improvement in Eczema Area and Severity Index-90 (EASI-90) among patients with moderate to severe atopic dermatitis. Nine randomized controlled trials (RCTs) were included, with 1231 patients in the lebrikizumab group and 658 in the placebo group. Lebrikizumab is shown to likely increase EASI-90 (RR 2.26, 95% CI 1.67–3.06, CoE Low), indicating that patients treated with lebrikizumab were more than twice as likely to achieve EASI-90 compared to those receiving placebo. Moderate heterogeneity was observed, with an I^2^ value of 56.8% and a τ^2^ of 0.0774, suggesting some variability between studies.

**Figure 8:**
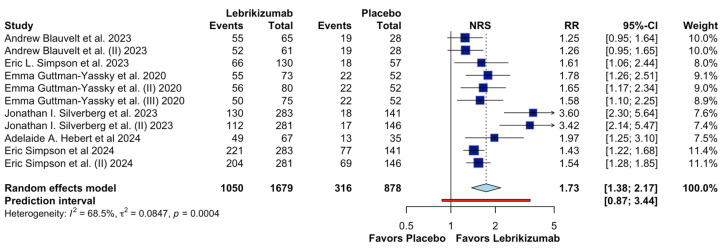
Effects of Lebrikizumab on NRS [4,9,13,14,17,18].

Figure 8 depicts the results of improvement in pruritus as measured by the Numerical Rating Scale (NRS) among patients with moderate to severe atopic dermatitis. Eleven randomized controlled trials (RCTs) were included, with 1679 patients in the lebrikizumab group and 878 in the placebo group. It is shown that lebrikizumab can slightly increase the NRS of pruritus (RR 1.73, 95% CI 1.38-2.17, low CoE), indicating that patients treated with lebrikizumab were 73% more likely to experience significant relief of pruritus compared to those receiving placebo. Moderate heterogeneity was observed between studies, with an I^2^ value of 68.5% and a τ^2^ of 0.0847, reflecting some variability in effect sizes between trials.

**Figure 9:**
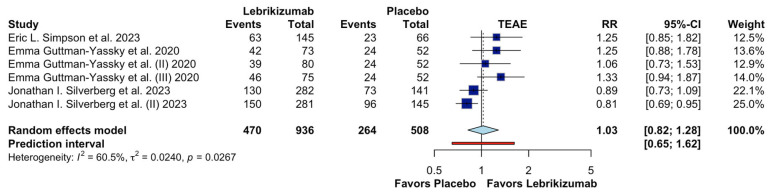
Effects of Lebrikizumab on TEAE [9,13,14].

Figure 9 summarizes the meta-analysis evaluating the risk of treatment-emergent adverse events (TEAEs) associated with lebrikizumab compared with placebo in patients with moderate-to-severe atopic dermatitis. Six randomized controlled trials were included, comprising a total of 936 patients in the lebrikizumab group and 508 patients in the placebo group.

The pooled analysis using a random-effects model showed no statistically significant difference in the risk of TEAEs between lebrikizumab and placebo (RR 1.03, 95% CI 0.82–1.28). This finding indicates that treatment with lebrikizumab does not appear to increase the overall incidence of treatment-emergent adverse events compared with placebo.

Moderate heterogeneity was observed across studies (I^2^ = 60.5%, τ^2^ = 0.0240, *p* = 0.0267), suggesting some variability in reported TEAE rates between trials. This heterogeneity may be explained by differences in study design, background therapy (monotherapy versus combination with topical corticosteroids), follow-up duration, and trial populations, including variations in disease severity and age distribution. Despite this variability, the direction of effect was generally consistent across studies, with individual trial estimates clustering around the null effect.

The prediction interval was relatively wide (RR 0.65–1.62), reflecting between-study variability and indicating that, in some future settings, lebrikizumab could be associated with either a lower or higher incidence of TEAEs compared with placebo. However, the absence of a significant pooled effect supports the overall conclusion that lebrikizumab does not confer an increased global safety risk in terms of TEAEs within the duration of the included randomized trials.

### 3.5. GRADE Assessment

The certainty of evidence for all outcomes was assessed using the GRADE framework, considering risk of bias, inconsistency, indirectness, imprecision, and publication bias on a domain-by-domain basis. The resulting certainty ratings reflect the overall confidence in the pooled effect estimates, integrating statistical findings with clinical and methodological considerations.

For the primary outcome (EASI-50), the certainty of evidence was rated as moderate. The included randomized controlled trials were judged to have a generally low risk of bias, with adequate randomization procedures and outcome assessment. No serious inconsistency was identified, as the pooled analysis demonstrated no heterogeneity (I^2^ = 0%). The population, intervention, comparator, and outcome definitions were directly applicable to the review question, and effect estimates were sufficiently precise. Publication bias could not be formally assessed due to the limited number of studies; however, there was no strong indication to downgrade on this domain.

For EASI-75, the certainty of evidence was rated as low. Although the pooled analysis showed a statistically significant benefit of lebrikizumab compared with placebo, substantial heterogeneity was observed (I^2^ = 77.5%). Predefined subgroup analyses based on dosing regimen and dosing frequency were conducted to explore potential sources of variability; however, residual heterogeneity persisted and no statistically significant subgroup interactions were identified. Consequently, the certainty of evidence was downgraded by one level for serious inconsistency. No additional downgrades were applied for risk of bias or indirectness, but publication bias could not be reliably assessed due to the small number of studies.

For EASI-90, the certainty of evidence was also rated as low. Moderate heterogeneity was observed across studies (I^2^ = 56.8%), indicating variability in effect estimates. In addition, imprecision contributed to uncertainty, as confidence intervals were relatively wide and the number of contributing events was limited. Taken together, these considerations justified downgrading the certainty of evidence, despite the consistent direction of effect favoring lebrikizumab.

For pruritus improvement measured by the Numeric Rating Scale (NRS), the certainty of evidence was rated as low. Although lebrikizumab was associated with a statistically significant improvement in pruritus, moderate heterogeneity was present (I^2^ = 68.5%). Differences in outcome assessment timing, baseline disease severity, and trial design likely contributed to this variability. As with other outcomes, formal assessment of publication bias was not feasible due to the limited number of studies.

For the safety outcome of treatment-emergent adverse events (TEAEs), the certainty of evidence was rated as moderate. The pooled analysis demonstrated no statistically significant difference in the risk of TEAEs between lebrikizumab and placebo, with moderate heterogeneity (I^2^ = 60.5%). The direction of effect was consistent across studies, and no signal of increased overall adverse events was observed. However, the certainty of evidence was not rated as high due to heterogeneity and the inability to assess publication bias formally.

Overall, certainty ratings differed across outcomes because GRADE judgments were informed by the combined assessment of multiple domains rather than heterogeneity alone. Outcomes with minimal heterogeneity and adequate precision retained higher certainty, whereas outcomes with substantial unexplained heterogeneity or imprecision were downgraded accordingly. These assessments are transparently documented in the GRADE evidence profile tables provided in the Supplementary Materials.

## 4. Discussion

In this study, we found that lebrikizumab demonstrated significant clinical efficacy, which was evident in two aspects: on the one hand, the severity of the disease, measured in different ways, and on the other, the impact on the symptoms perceived by patients with moderate to severe atopic dermatitis. When compared to placebo, lebrikizumab therapy significantly increased the likelihood of achieving EASI-75, EASI-50, and EASI-90 responses. Similarly, a clinically significant improvement in pruritus, measured using the Numerical Rating Scale (NRS), was observed. All of the above supports the use of IL-13 inhibition therapy in patients with complicated disease [4,9,13,16,17,18].

However, substantial heterogeneity was observed in some key outcomes, particularly in EASI-75. In response, it was decided to perform predefined subgroup analyses related to dosing frequency (Q2W versus Q4W), given that several clinical trials of atopic dermatitis have shown that differences in dose magnitude, administration schedule, and timing of assessment are known sources of variability [4,9,13,15,17]. Although the subgroup analyses showed consistency in the direction of effect across all dosing regimens, no statistically significant interactions were identified between the subgroups evaluated, and residual heterogeneity persisted.

According to the GRADE recommendations, based on the available evidence, EASI-75 was assigned a low level of certainty due to inconsistency, as the substantial heterogeneity could not be explained, whereas the more consistent EASI-50 findings retained a moderate level of certainty [8,12].

From a mechanistic standpoint, lebrikizumab selectively inhibits interleukin-13 (IL-13), which plays a fundamental role in epidermal barrier dysfunction, type 2 inflammation, and the development of pruritus in atopic dermatitis [5,6,7]. Experimental and translational studies have shown that IL-13 blockade reduces inflammatory infiltration and, consequently, improves cutaneous barrier integrity; this has become the biological basis for the clinical benefits observed in randomized trials [5,6]. Accordingly, IL-13 inhibition represents a rational and targeted therapeutic approach for moderate-to-severe atopic dermatitis, with efficacy supported by multiple clinical assessment endpoints [7,13].

With respect to the safety profile of lebrikizumab, the available evidence generally suggests that it is well tolerated. Across phase II and III trials, the most frequently reported adverse events were mild to moderate, such as injection-site reactions and conjunctivitis, and no treatment-related serious adverse events or therapy discontinuations were identified [4,9,13,16,17,18]. However, interpretation of safety outcomes should remain cautious, as follow-up duration differed across studies and randomized trials may therefore have failed to detect rare or long-term adverse effects, underscoring the importance of post-marketing pharmacovigilance and real-world data [19].

The characteristics of the individual trials may help explain the observed variability. For example, follow-up duration varied widely, ranging from 12 to 52 weeks, and outcomes were assessed at different phases of treatment, such as induction versus maintenance [4,9,17,18]. In addition, adolescents accounted for less than 18% of participants, which limits the generalizability of pooled estimates; where age distributions differed across studies, this may also have contributed to the heterogeneity observed [17,18].

Although concomitant topical corticosteroid use represents a clinically plausible source of heterogeneity, a formal subgroup analysis comparing lebrikizumab mono-therapy versus combination therapy with topical corticosteroids could not be reliably performed. Across the included trials, background topical therapy was inconsistently reported, and definitions of combination therapy varied substantially, precluding harmonized extraction of comparable subgroups. This limitation highlights a recurrent challenge in meta-analyses of atopic dermatitis trials and underscores the need for standardized reporting of concomitant therapies in future studies [9,15,19].

Overall, the findings of this study are broadly consistent with prior randomized clinical trials and systematic reviews evaluating IL-13 inhibition in atopic dermatitis. Nonetheless, they also highlight important sources of uncertainty related to outcome heterogeneity, the composition of the populations studied, and trial design [4,9,13,15,16,17,18,19]. These factors should be taken into account not only when interpreting the results in clinical practice, but also when designing future studies from a clinician’s standpoint, the marked efficacy of lebrikizumab in reducing pruritus is a major advantage, especially since patients consistently rate itch as their most burdensome symptom. Moreover, because the drug selectively targets IL-13 and avoids the decoy receptor, it theoretically minimizes disruption to the body’s natural regulatory processes. While our meta-analysis confirms its short-term efficacy and safety, it remains to be seen whether this specific mechanism yields better outcomes than broader type 2 blockade in real-world practice over time.

### Strengths and Limitations

Key strengths of this study include a comprehensive search strategy across multiple databases, adherence to PRISMA-2020 reporting guidelines, the use of random-effects meta-analytic models, and a systematic assessment of the certainty of evidence using the GRADE framework [8,12]. Taken together, these methodological choices strengthen the transparency of our process and support the reproducibility of our findings.

At the same time, several limitations should be acknowledged. A formal evaluation of publication bias was not possible because only a small number of studies were available for most outcomes. We observed moderate-to-high heterogeneity for key endpoints—particularly EASI-75 and pruritus—which complicates interpretation and may limit generalizability to broader clinical settings. In addition, the underrepresentation of participants younger than 18 years reduces the confidence of conclusions for this subgroup. Finally, differences in dosing regimens, follow-up duration, and the reporting of concomitant topical therapies likely contributed to residual heterogeneity that could not be fully accounted for through subgroup analyses [9,15,17,18,19].

## 5. Conclusions

Lebrikizumab shows clinically meaningful benefits over placebo in reducing both overall disease severity and pruritus in patients with moderate-to-severe atopic dermatitis, while maintaining a generally favorable safety profile in randomized trials. However, the substantial heterogeneity observed in EASI-75 outcomes and the limited representation of adolescents call for cautious interpretation of pooled effect estimates. Further research—featuring more harmonized trial designs, standardized reporting of concomitant therapies, and more inclusive study populations—is needed to better define the drug’s efficacy and safety as it is used in routine clinical practice [4,9,15,17,18,19,20].

## Figures and Tables

**Table 1:** Characteristics of included studies.

Authors	Year of Publication	Country of Origin	Study Type	Study Phase	Number of Centers and Locations
Eric L. Simpson et al. [16]	2018	Multinational	A randomized, placebo-controlled, double-blind	Phase 2	62 centers
Emma Guttman-Yassky et al. [13]	2020	USA	Randomized, double-blind, placebo-controlled, dose-ranging clinical trial	Phase 2b	57 US centers
Andrew Blauvelt et al. 14]	2023	Multinational	Randomized, double-blinded, placebo-controlled	Phase III	171 study sites in North America, Europe, Asia-Pacific
Eric L. Simpson et al. [9]	2023	Germany, Poland, Canada, USA	Randomized, double-blind, placebo-controlled clinical trial	Phase 3	54 outpatient sites
Jonathan I. Silverberg et al. [14]	2023	Multinational	Randomized, double-blind, placebo-controlled, parallel-group clinical trial	Phase 3	NR
Akio Tanaka et al. [15]	2024	Multinational	Randomized placebo-controlled clinical trial	Phase 3	37 centres
Adelaide A. Hebert et al. [17]	2024	US; Europe; Rest of world	Three randomized, double-blind, placebo-controlled	Phase 3	NR
Eric Simpson et al. [18]	2025	US; Europe; Rest of world	Two randomized, double-blind, placebo-controlled	Phase 3	100 study sites in the United States, Canada, Europe, and the Asia–Pacific

**Table 2:** Characteristics of patients included.

Authors	Year of Publication	Sample Size (Total, by Intervention and Control Groups)	Average Age and Range	Gender Distribution	Weight and Height	Comorbidities Reported
Andrew Blauvelt et al. [4]	2023	Total: 806; Intervention (Q2W): 113; Intervention (Q4W): 118; Placebo: 60	Mean age: 35.5 years; Range: ≥12 years	Female: 36% (withdrawal), 58.5% (Q4W), 46.9% (Q2W)	Mean weight: 72.7 kg (withdrawal), 74.6 kg (Q4W), 76.2 kg (Q2W)	Asthma and rhinitis
Eric L. Simpson et al. [9]	2023	Total: 211, LEB + TCS: 145, PBO + TCS: 66	Mean age: 37.2 years (SD: 19.3)	Female: 48.8%	Mean weight: 74.6 kg (LEB + TCS)	None specified
Emma Guttman-Yassky et al. [13]	2020	Total: 280, Placebo: 52, Lebrikizumab groups: 228	Mean age: 39.3 years (SD: 17.5)	Female: 59.3%	BMI mean: 29.7 (placebo), 30.1, 29.2, 28.1 (Lebrikizumab groups)	None specifically listed
Jonathan I. Silverberg et al. [14]	2023	Trial 1: lebrikizumab (283), placebo (141) Trial 2: lebrikizumab (281), placebo (146)	Trial 1: Placebo (34.2 ± 16.4) range: 12 to <18 years: 18 (12.8), ≥18 years: 123 (87.2), Lebrikizumab (36.1 ± 17.8) range: 12 to <18 years: 37 (13.1), ≥18 years: 246 (86.9) Trial 2: Placebo (35.3 ± 17.2) range: 12 to <18 years: 17 (11.6), ≥18 years: 129 (88.4), Lebrikizumab (36.6 ± 16.8) range: 12 to <18 years: 30 (10.7), ≥18 years: 251 (89.3).	Trial 1: placebo 73 (51.8), lebrikizumab 141 (49.8) Trial 2: placebo 75 (51.4), lebrikizumab 136 (48.4)	WEIGHT: Trial 1: placebo (79.0 ± 22.7), lebrikizumab (77.0 ± 19.7) Trial 2: placebo (76,0 ± 21.1), lebrikizumab (76.7 ± 20.5)	NR
Akio Tanaka et al. [15]	2024	286 patients, including 18 adolescents, were randomized 2:2:3 to receive placebo (82 patients), lebrikizumab Q4W (81 patients), and lebrikizumab Q2W (123 patients) in combination with TCS	Placebo + TCS: 34.8 (13.6), Adults (≥18 years): 76 (92.7), Adolescents (≥12 to <18 years): 6 (7.3), Lebrikizumab Q4W + TCS: 37.8 (12.0), Adults (≥18 years): 77 (95.1) Adolescents (≥12 to <18 years): 4 (4.9), Lebrikizumab Q2W + TCS: 35.5 (12.2): Adults (≥18 years): 115 (93.5), Adolescents (≥12 to <18 years): 8 (6.5)	Female, n (%): Placebo + TCS: 24 (29.3), Lebrikizumab Q4W + TCS: 25 (30.9), Lebrikizumab Q2W + TCS: 41 (33.3)	Weight, kg, mean (SD): Placebo + TCS: 65.6 (12.5), Lebrikizumab Q4W + TCS: 66.5 (13.8), Lebrikizumab Q2W + TCS: 64.9 (11.4), <60 kg: Placebo + TCS: 29 (35.4), Lebrikizumab Q4W + TCS: 29 (35.8), Lebrikizumab Q2W + TCS: 47 (38.2), ≥60 to <100 kg: Placebo + TCS: 53 (64.6), Lebrikizumab Q4W + TCS: 51 (63.0), Lebrikizumab Q2W + TCS: 76 (61.8), ≥100 kg: Placebo + TCS: 0, Lebrikizumab Q4W+TCS: 1 (1.2), Lebrikizumab Q2W + TCS: 0	Hand dermatitis, Facial dermatitis, Conjunctivitis, Herpes zoster, Chickenpox, Eczema herpeticum, Herpetic whitlow, Disseminated neonatal herpes simplex, Herpes labialis, Genital herpes, Asthma, Alopecia, Food allergy, Allergic rhinitis, Pollinosis
Eric L. Simpson et al. [16]	2018	**Total patients: 209 patients** (1) Lebrikizumab 125 mg single dose: 52 (2) Lebrikizumab 250 mg: 53 (3) Lebrikizumab 125 mg Q4W: 51 (4) Placebo: 53	Range: 18-75 years old Mean Age: (1) Lebrikizumab 125 mg single dose: 34.9 (SD: 12.7) (2) Lebrikizumab 250 mg: 34.4 (12.3) (3) Lebrikizumab 125 mg Q4W: 36.6 (12.3) (4) Placebo: 38.7 (13.2)	Male %: (1) Lebrikizumab 125 mg single dose: 65.4% (2) Lebrikizumab 250 mg: 58.5% (3) Lebrikizumab 125 mg Q4W: 68.6% (4) Placebo: 67.9%	NR	NR
Adelaide A. Hebert et al. [17]	2024	**Total patients:148** lebrikizumab (N = 67) vs. placebo (N = 35); (lebrikizumab + TCS, N = 32; placebo + TCS, N = 14	≥12 to <18 years weighing ≥40 kg	Female: 54.7%	≥40 kg	NR
Eric Simpson et al. [18]	2024	**Total patients: 869** LEB 250 mg Q2W, N = 564 PBO, N = 287	adults (aged ≥18 years) and adolescents (aged 12 to <18 years and weighing ≥ 40 kg)	Female: 50.5%	≥40 kg	NR

**Table 3:** Characteristics of intervention studies.

Authors	Year	Intervention Strategy	Dose and Schedule	Treatment Period	Concomitant Therapy	Follow-Up	Attrition	Adherence
Blauvelt et al. [4]	2023	Lebrikizumab vs. placebo	250 mg Q2W or Q4W	52 weeks (16-week induction + 36-week maintenance)	Intermittent topical corticosteroids permitted	52 weeks	≤12.9% across arms	High
Simpson et al. [9]	2023	Lebrikizumab + TCS vs. placebo + TCS	Loading 500 mg, then 250 mg Q2W	16 weeks	Low–mid potency topical corticosteroids	16 weeks	19 patients	High
Guttman-Yassky et al. [13]	2020	Lebrikizumab vs. placebo	125 mg or 250 mg Q2W/Q4W	16 weeks	Rescue therapy allowed	16 weeks (+safety)	Completion: 44.2% placebo; ~77–80% lebrikizumab	High
Silverberg et al. [14]	2023	Induction–maintenance lebrikizumab vs. placebo	Induction: 500 mg loading + 250 mg Q2W; Maintenance: Q2W or Q4W	52 weeks	Topical corticosteroids as rescue	36 weeks	≤1.4%	NR
Tanaka et al. [15]	2024	Lebrikizumab + TCS vs. placebo + TCS	250 mg Q2W or Q4W with loading dose	68 weeks (incl. safety)	Low–mid potency TCS and/or TCI	52 weeks	1.4%	98.6% completed induction
Simpson et al. [16]	2018	Lebrikizumab vs. placebo	125 mg single dose; 250 mg; 125 mg Q4W	12 weeks	Medium-potency TCS	12 weeks	NR	NR
Hebert et al. [17]	2024	Lebrikizumab ± TCS vs. placebo	Loading 500 mg, then 250 mg Q2W	16 weeks	TCS optional (tapering allowed)	NR	NR	High
Simpson et al. [18]	2024	Lebrikizumab monotherapy vs. placebo	250 mg Q2W	16 weeks	None	52 weeks	NR	High

## Data Availability

Data are contained within the article.

## Supplementary Materials

The following supporting information can be downloaded at: https://www.mdpi.com/article/10.3390/jcm15051737/s1, Table S1: Search strategy. Table S2: PRISMA 2020 checklist.
