# Supplementary material for: Efficacy and Safety of Lebrikizumab in Adults with Moderate-to-Severe Atopic Dermatitis: A Systematic Review and Meta-Analysis
Source: J Clin Med. 2026 Feb 25;15(5):1737. doi: 10.3390/jcm15051737 (PMC12986258; doi:10.3390/jcm15051737)
Supplement: Supplementary file 1 [file jcm-15-01737-s001.zip › jcm-4018458-supplementary/Table S1.pdf]

Supplementary table S1. Search strategy

Engine	Strategy	Results
Pubmed	#1 = ("Atopic Dermatitis" OR "Eczema, Atopic" OR "Atopic Eczema" OR "Neurodermatitis, Atopic" OR "Atopic Neurodermatitis" OR "Neurodermatitis, Disseminated" OR "Disseminated Neurodermatitis" OR "Eczema, Infantile" OR "Infantile Eczema")	102
	#2= ("Lebrikizumab")	
	#4= #1 AND #2	
Scopus	#1= TITLE-ABS-KEY ("Atopic Dermatitis" OR "Eczema, Atopic" OR "Atopic Eczema" OR "Neurodermatitis, Atopic" OR "Atopic Neurodermatitis" OR "Neurodermatitis, Disseminated" OR "Disseminated Neurodermatitis" OR "Eczema, Infantile" OR "Infantile Eczema")	327
	#2= TITLE-ABS-KEY (Lebrikizumab)	

Web of Science	#1 = ("Atopic Dermatitis" OR "Eczema, Atopic" OR "Atopic Eczema" OR "Neurodermatitis, Atopic" OR "Atopic Neurodermatitis" OR "Neurodermatitis, Disseminated" OR "Disseminated Neurodermatitis" OR "Eczema, Infantile" OR "Infantile Eczema")	171
	#2= (Lebrikizumab)	
Embase	#1 = ('dermatitis'/exp OR dermatitis OR 'atopic dermatitis')	434
	#2 = 'lebrikizumab'	
	TOTAL SEARCH	1034
	DUPLICATES	506
	TOTAL REGISTERS AFTER DUPLICATES REMOVED	528
